# Molecular and Epigenetic Mechanisms Underlying Cognitive and Adaptive Responses to Stress

**DOI:** 10.3390/epigenomes1030017

**Published:** 2017-11-02

**Authors:** Alexandra F. Trollope, Karen R. Mifsud, Emily A. Saunderson, Johannes M. H. M. Reul

**Affiliations:** 1 Neuro-Epigenetics Research Group, Bristol Medical School, University of Bristol, Dorothy Hodgkin Building, Whitson Street, Bristol BS1 3NY, UK; 2 Department of Anatomy, College of Medicine and Dentistry, James Cook University, Townsville 4811, Australia; 3 Barts Cancer Institute, Queen Mary University of London, London EC1M 6BQ, UK

**Keywords:** epigenetics, cognition, glucocorticoid hormone, stress, memory, learning, hippocampus, glucocorticoid receptor, mineralocorticoid receptor, immediate-early gene

## Abstract

Consolidation of contextual memories after a stressful encounter is essential for the survival of an organism and in allowing a more appropriate response to be elicited should the perceived threat reoccur. Recent evidence has explored the complex role that epigenetic mechanisms play in the formation of such memories, and the underlying signaling pathways are becoming more apparent. The glucocorticoid receptor (GR) has been shown to play a key role in these events having both genomic and non-genomic actions in the brain. GR has been shown to interact with the extracellular signal-regulated kinase mitogen-activated protein kinase (ERK MAPK) signaling pathway which, in concert, drives epigenetic modifications and chromatin remodeling, resulting in gene induction and memory consolidation. Evidence indicates that stressful events can have an effect on the offspring in utero, and that epigenetic marks altered early in life may persist into adulthood. A new and controversial area of research, however, suggests that epigenetic modifications could be inherited through the germline, a concept known as transgenerational epigenetics. This review explores the role that epigenetic processes play in the central nervous system, specifically in the consolidation of stress-induced memories, the concept of transgenerational epigenetic inheritance, and the potential role of epigenetics in revolutionizing the treatment of stress-related disorders through the emerging field of pharmacoepigenetics and personalized medical treatment.

## 1 Introduction

The stress response is initiated when an animal encounters a perceived harmful event or situation, physical and/or psychological, that threatens to disrupt homeostasis and requires appropriate physiological and behavioral responses in order to cope with the stressor [1,2]. A physical challenge involves the minor cognitive interpretation of the event and results in an immediate physiological response. In contrast, a psychological stressor requires the animal to assess the situation and make a decision through cognitive evaluations. These stressors evoke neurobiological changes, which result in behavioral adaption to increase the animal’s chance of survival [3,4]. Furthermore, memory formation of stressful events is required so that an individual will respond more fittingly should the stressful situation be encountered again. It should be noted that most stressful situations, if not all, are mixed in their physical versus psychological nature.

The body responds to stressors through mobilization of different physiological pathways including fast activation of the sympathetic nervous system, including the sympathoadrenomedullary system (SAS), and the slower hypothalamic-pituitary-adrenal (HPA) axis. SAS activation results in immediate release of adrenaline from the adrenal medulla into the systemic circulation to prepare the animal for the ‘fight or flight’ response. Activation of the HPA axis results in the secretion of glucocorticoid hormones (GCs; predominantly corticosterone in rodents such as rats and mice, cortisol in humans) from the adrenal cortex into the circulation.

GCs bind to corticosteroid receptors, i.e., the mineralocorticoid receptors (MRs (encoded by the *Nr3c2* gene) and GC receptors (GRs; *Nr3c1*)), that co-localize in parts of the limbic system, i.e., the hippocampus [5–7]. The molecular role that GCs play in producing long-lasting behavioral changes appears to be highly complex involving both genomic and non-genomic mechanisms. Classically, as part of their genomic action, MRs and GRs act as ligand-dependent transcription factors that control the expression of GC target genes through interaction with GC-response elements (GREs) located throughout the genome often within or in the vicinity of these genes [8–10]. Non-genomic effects have been demonstrated regarding membrane-bound MRs and GRs, resulting in neurophysiological changes [11] and, through the interactions of GRs with intracellular signaling pathways such as the extracellular signal-regulated kinase mitogen-activated protein kinase (ERK MAPK) pathway in the hippocampus, resulting in epigenetic and genomic changes, and behavioral adaptation [12,13].

The term epigenetics was first coined by Waddington in 1942 to describe phenotype development [14]. We now know that although cells all contain the same genetic information they display a rich variety of phenotypes, varying greatly in morphology and function. This is now recognized as the origin of the differential expression of genes that occurs without changes to the DNA sequence. Furthermore, epigenetics has been regarded as the ‘molecular bridge’ between the cell’s genotype and the potentially ever-changing phenotype.

This review will explore the role of epigenetic and molecular mechanisms in the central nervous system (CNS) covering current mechanisms underpinning stress-related learning and memory, transgenerational epigenetics, the role epigenetics plays in neuropsychiatric disorders, and the potential of using epigenetic modifications as biomarkers, or to inform the most effective course of treatment.

## 2 Epigenetics

Epigenetic mechanisms determine the way genes are organized in the cell nucleus and influence their expression by changing the conformation of the chromatin and therefore the accessibility of the DNA for transcription factors, other factors, and the transcriptional machinery. These epigenetic mechanisms include post-translational histone modifications (PTMs), DNA methylation, and non-coding RNAs, resulting in activation, silencing, or poising of genes and thereby regulating patterns of gene expression. Epigenetic mechanisms are highly dynamic and allow cells to respond to changes in their environment, thereby contributing to the plasticity of the brain and thus the way the brain responds to environmental challenges such as stress and, as a consequence, facilitating learning and memory [15]. Learning and the formation of memories require gene transcription and protein synthesis in vivo [16], which, amongst others, contribute to the structural and functional remodeling of synapses between neurons [17]. Recent evidence suggests that these epigenetic modifications may also be carried across generations, which has instigated a new area of research termed transgenerational epigenetic inheritance.

### 2.1 Epigenetic Mechanisms in Stress-Related Learning and Memory Paradigms

Epigenetic mechanisms play a key role in how animals consolidate memories associated with a stressful event. It should be noted that any behavioral test imposed on rodents will represent a stressful situation for such animals. Therefore, stress is an integral part of behaviorally relevant challenges to these rodents. Examples of such behavioral challenges are contextual fear conditioning, Morris water maze (MWM) learning, and the forced swim (FS) test [4,12,13,18]. The different types of PTMs can be addressed individually in stress-related learning and memory paradigms (as below); however, it should be considered that many PTMs work together in order to direct gene transcription and that this can be in a tissue/cell-specific manner.

#### 2.1.1 Acetylation

Acetylation of specific histones and/or specific residues has been found to be associated with long-term memory formation. For example, after contextual fear conditioning, histone H3 but not H4 acetylation increased specifically within the CA1 region of the hippocampus, which was dependent of the activation of *N*-methyl-D-aspartate (NMDA) receptors (NMDA-Rs) and, subsequently, the ERK MAPK signaling pathway [19]. Long-term memory was enhanced upon the use of the HDAC inhibitor NaBut (sodium butyrate) in vivo prior to contextual fear conditioning [19]. In MWM studies, increased acetylation of histone H4 at lysine 12 (H4K12ac) and pan acetylation of H2B in the hippocampus were found to be specifically associated with spatial learning [20]. This observation demonstrates that distinct histones are subject to selective modifications due to a behavioral challenge. Furthermore, recent studies have shown that epigenetic modifications can vary not only between different regions of the brain but also between the sub-regions. For example, Castellano and colleagues [21] discovered that after training in a one-day redundant place/cue version of the MWM there was an increase in pan-acetylated H3 and H4 and a decrease in the acetylation of H3 lysine 9 (H3K9) within the CA1 sub-region. In parallel, in the CA3 region only H3 pan-acetylation was increased, whereas in the dentate gyrus (DG) pan-acetylation of H3 and phosphorylation of H3 at serine 10 (H3S10p) occurred in a small subset of DG granular neurons [22]. These experiments further demonstrate the complexity of the epigenetic histone modifications.

#### 2.1.2 Phosphorylation

In the FS test the behavioral immobility response observed 24 h or even several weeks after the initial test is an adaptive behavioral response that depends on a GR-mediated action of GC hormones in the DG of the hippocampus [4,13,23–25]. We found, serendipitously, that forced swimming raises histone H3 phosphorylation (H3S10p) selectively in sparsely distributed, mature dentate granule neurons in rats and mice [26,27]. It appeared that this histone mark exists in combination with an acetylation mark at lysine14 (i.e., H3S10p-K14ac; and lysine9 H3K9ac-S10p; [12]), indicating that this combinatorial epigenetic mark could be involved in transcriptional activation [27,28]. Subsequent studies showed that the formation of H3S10p-K14ac in DG neurons was dependent on signaling through GRs and NMDA-Rs [13,27,28]. Furthermore, we established that a crucial link between forced swimming-induced NMDA-R activation and the formation of H3S10p-K14ac in DG neurons is the phosphorylation of ERK1/2 (by MAPK/ERK kinase (MEK)) and the recruitment of the nuclear kinases MSK1/2 (mitogen- and stress-activated kinases 1 & 2) and ELK1 (ETS transcription factor 1) [13]. Previously, it has been demonstrated in vitro that the kinases ERK1/2 activate MSK1 [29] and ELK1 [30,31] through phosphorylation. Studies in vitro have also shown that the ERK MAPK pathway can activate Elk-1 through phosphorylation and activation of cAMP response element binding protein (CREB) [31,32]. MSK1/2 is a H3S10 kinase, whereas Elk-1 can recruit the histone acetyl-transferase p300, which can acetylate histone H3 at various lysine residues. Importantly, it was found that activation of the NMDA-R/ERK1/2/MSK1/2-ELK1 signaling pathway was critical for the consolidation of the behavioral immobility response [13,27,33]. Inspired by the pioneering work of Clayton et al. [34], the role of the immediate early genes (IEGs) *Fos* and *Egr1* in the FS paradigm was investigated. These IEGs are involved in long-term memory paradigms like the MWM [35]. Based on a series of pharmacological, gene deletion, immunohistochemical, and chromatin immunoprecipitation (ChIP) studies, it was indeed found that the formation of H3S10p-K14ac is associated with the induction of FOS and EGR1 in DG neurons [12,13,27,28]. Moreover, the induction of these IEGs in DG neurons was also found to be dependent of signaling through GRs [13].

**Figure 1:**
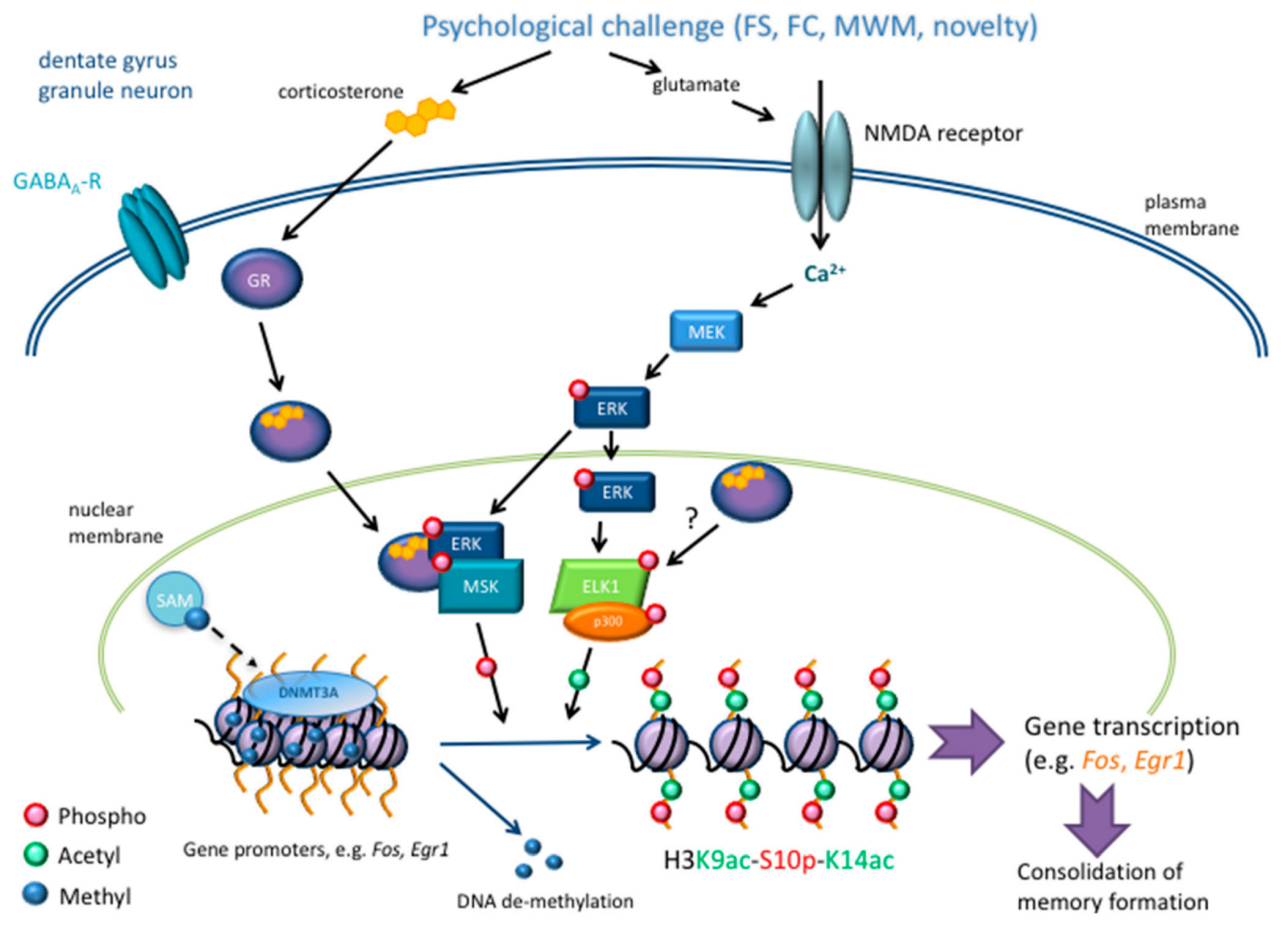
Psychological stress-evoked signaling and epigenomic responses in sparse dentate gyrus neurons underpinning gene transcription and contextual memory consolidation. The stressful challenge associated with forced swimming, Morris water maze learning, contextual fear conditioning, and novelty results in the activation of NMDA-R-ERK-MAPK pathways and GRs which, in conjunction, results in the activation of nuclear MSK1 and ELK1/p300. The activation of this histone kinase and histone acetyl-transferase leads to the formation of the combinatorial H3K9ac-S10p–K14ac histone marks within the promoter regions of the immediate-early genes *Fos* and *Egr1*, thereby facilitating the induction of gene transcription. Immediate-early gene induction is of critical importance for the consolidation of (contextual) memories associated with the stressful event. The recruitment of DNA methyltransferase 3A (DNMT3A) in conjunction with the concentration of the endogenous methyl donor SAM plays an important role in the DNA methylation state of the 5′-UTRs and promoter regions of the immediate-early genes *Fos* and *Egrl*, thereby controlling their expression as well as the consolidation of memories. See text for further details and literature references. FS: forced swimming; FC: contextual fear conditioning; MWM: Morris water maze training.

The dependency of IEG induction and the behavioral immobility response of both GRs and NMDA-R/ERK1/2/MSK1/2-Elk-1 signaling prompted the question about the biochemical basis of this confluence of molecular pathways [13,36]. Based on co-immunoprecipitation and other studies we found that GRs facilitate the phosphorylation (i.e., activation) of MSK1/2 and ELK1 through a fast protein-protein interaction with pERK1/2 [13]. These observations explained how the GR antagonist impaired the phosphorylation of MSK1/2 and ELK1, resulting downstream in a decline in both H3S10p-K14ac formation and IEG induction, without affecting ERK1/2 phosphorylation [13]. For an overview of these interacting signaling and epigenetic pathways, see Figure 1. The interaction of GRs with ERK1/2/MSK1/2-ELK1 signaling presents a novel non-genomic mechanism, which is quite distinct from its classical genomic mode of action (Figure 1).

After the discovery of the role of the NMDA-R/ERK/MAPK signaling pathway in evoking epigenetic (H3S10p-K14ac) changes and induction of IEGs in the FS and novelty tests, it was hypothesized that a similar sequence of events may be taking place in the MWM. It is also well-known that MWM learning is highly dependent on NMDA-R and ERK MAPK signaling [37,38]. Furthermore, deletion of MSK1 had been shown to result in impaired MWM learning and contextual fear learning, as well as a decrease in histone acetylation and phosphorylation after learning [39]. We found a positive correlation between the average latency to find the platform and the number of H3S10p-K14ac-positive DG neurons. Moreover, we found a significant increase of H3K9ac-S10p formation at the promoters of the *Fos* and *Egr1* genes but not at the *Arc* gene promoters in rats that had undergone MWM training, compared with baseline controls [22].

#### 2.1.3 Histone and DNA Methylation

Contextual fear conditioning experiments have been instrumental in elucidating the potential role that the complex PTMs at histones involving methylation may play in learning and memory. Tri- and di-methylation of H3K4 (H3K4me3 and H3K4me2, respectively) were increased after contextual fear conditioning in the CA1 sub-region of the hippocampus [40]. Specifically, H3K4me3 increased at the transcriptionally active *Egr1* and brain-derived neurotrophic factor (*Bdnf*) genes after contextual fear conditioning. Interestingly, however, DNA methylation also increased at the promoter of the *Egr1* gene but decreased at the *Bdnf* promoter of transcript 1 [40]. These findings are intriguing since DNA methylation is thought of as a transcriptional repressor and therefore was expected to reduce gene transcription [40]. It was suggested that MeCP2 could be binding to the methylated DNA along with CREB1, which can actively regulate gene transcription as demonstrated by Chahrour and colleagues [41].

Generally, it is thought that DNA methylation is associated with condensed and compacted chromatin, shielding binding sites from their transcription factors, and resulting in gene repression. DNA demethylation is thought to have the opposite effect, allowing transcriptional activation, which is essential for synaptic plasticity and learning and memory. This straightforward concept was supported by early studies demonstrating that depolarization of neurons in vitro results in a reduction of DNA methylation of the *Bdnf* gene and an increase in gene transcription [42]. Three functional enzymes responsible for DNA methylation in mammals have been identified, namely DNA methyltransferase 1 (DNMT1), DNA methyltransferase 3A (DNMT3A), and DNA methyltransferase 3B (DNMT3B) [43]. Based on the notion that DNA methylation results in gene silencing, it was assumed that DNMT inhibitors would lead to enhanced synaptic plasticity and potentially enhance learning and memory. Interestingly, pretreatment with DNMT inhibitors Zebularine and 5-aza-2′-deoxycytidine (5-Aza) resulted in blockade of synaptic plasticity [44]. These experiments showed that long-term potentiation may be affected by both activation and inhibition of gene transcription. These surprising findings may, at least in part, be related to the recently reported observations that certain DNMTs can act as DNA methyltransferases as well as DNA demethylases, depending on the cell activation status and methyl donor concentration [45–47].

DNA methylation changes and DNMT expression have been investigated in various animal models. Chronic social defeat stress was shown to induce prolonged anxiety-like behavior and to result in a reduction of DNMT3A mRNA levels and global DNA methylation levels in the medial prefrontal cortex (mPFC). DNMT3A knock-down in the mPFC resulted in enhanced anxiety-like behavior similar to mice that had undergone chronic social defeat stress, while over-expression resulted in a reduction of anxiety levels [48]. It is well-known that there is a higher prevalence of depression and anxiety disorders in females than in males and the symptoms displayed are different as well [49,50]. Hodes et al. studied the neurobiological basis of these sex differences in an animal model using sub-chronic variable stress. Using this model, they demonstrated sex differences in the transcriptomic profile in the Nucleus Accumbens, which appeared to be associated with susceptibility versus resilience to sub-chronic variable stress. Data indicated that DNMT3A may play an important role in the mRNA expression changes observed in this brain reward region [51].

**Figure 2:**
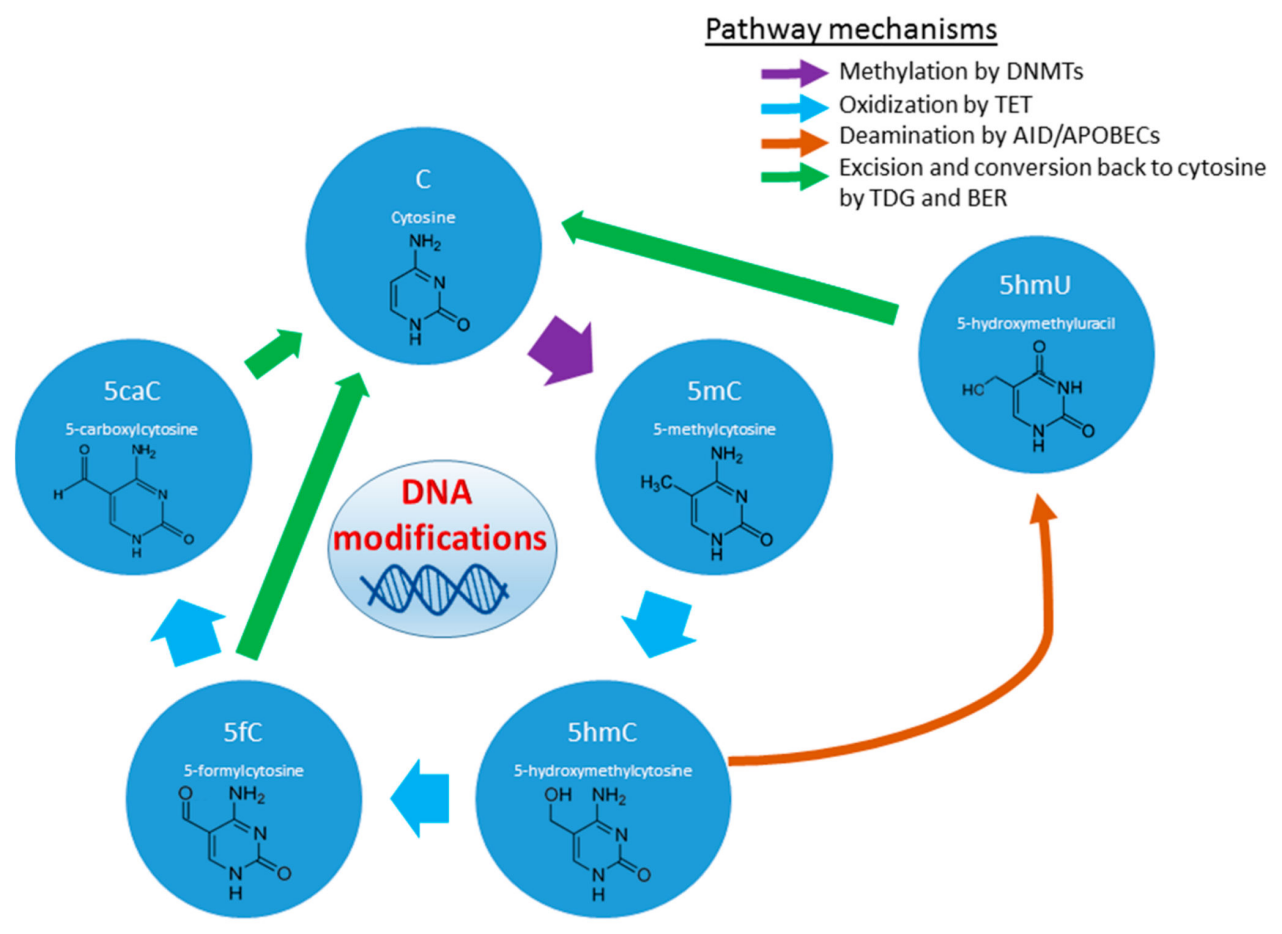
Epigenetic modifications of DNA. Cytosines within the DNA sequence can be dynamically modified into stable forms which may have specific functional roles. Abbreviations: DNA methyltransferases (DNMTs), ten-eleven translocation (TET) enzymes, activation-induced cytidine deaminase (AID), apolipoprotein B mRNA-editing enzyme catalytic polypeptides (APOBEC), thymine DNA glycosylase (TDG), base excision repair (BER) pathway.

Furthermore, recent research has uncovered the true complexity of the range of modifications occurring at these ‘methylation’ sites on DNA [52] (Figure 2). Various intermediate modifications may have their own specific regulatory control modalities on gene transcription. One of these intermediate marks, 5-formylcytosine (5fC), derived from ten-eleven translocation gene protein 1 (TET1)-mediated oxidation of 5-hydroxymethylation (5-hmC), has been shown to be associated with the recruitment of the transcription factor ING1 (inhibitor of growth family member 1). This association has been linked to the transcription of essential genes within the mPFC that are required for successful fear extinction training in mice [53].

Lubin and colleagues showed that contextual fear conditioning induced differential regulation of exon-specific Bdnf mRNA transcripts in the hippocampus, which were associated with changes in the *Bdnf* DNA methylation pattern [54]. BDNF is known to contribute to neuronal activity-dependent processes such as long-term potentiation [55]. Furthermore, DNA methylation appears to determine the *Bdnf* transcripts produced during fear memory consolidation [54]. Inhibition of DNMT action using the DNA methylation inhibitor Zebularine in rats resulted in *Bdnf* demethylation. This demethylation caused an associated increase in the output of *Bdnf* gene transcripts and, surprisingly, blocked memory consolidation [54]. Contrary to expectations, inhibiting DNMTs prevented DNA demethylation within specific sites of the *Bdnf* gene in rats [54]. As mentioned before, such effects can potentially be explained by DNA de-methylase activity of certain DNMTs; however, at the time of the above study [54], it was considered possible that the observed repression was mediated through a memory suppressor gene such as the protein phosphatase 1 (PP1) [56]. It has also been suggested that DNMTs can regulate both DNA methylation and demethylation via indirect mechanisms and/or pathways [57]. Fear conditioning is associated with an increase in the methylation specifically at the promoter of *PP1* and a decrease in DNA methylation at the promoter of the plasticity-associated gene *Reelin* [56]. These results show that DNA methylation and demethylation is clearly very dynamic, and both are essential for neuronal plasticity and memory consolidation. A recent study has shown for the first time that DNMT3B1 can be recruited to regions of active transcription, specifically in regions with elevated H3K36me3, which appeared to guide binding of DNMT3B1 and resulted in site-specific de novo methylation in mouse stem cells [58]. This study demonstrates that DNMT3B1 is binding to actively transcribed genes in a cell-type specific manner, resulting in de novo methylation and reduction of gene transcription. These experiments indicate that complex mechanisms control DNA methylation status, conferring the precise and dynamic nature of DNA methylation/demethylation processes. Miller and Sweatt also demonstrated that contextual fear conditioning results in an increase in de novo *Dnmt3a* and *Dnmt3b* gene expression in the hippocampus and that inhibition of the DNMTs blocked memory formation. Future work delineating the DNA methyl-transferase versus DNA demethylase activity status of DNMTs under various conditions of behavioral/neuronal activity will clarify the exact role of these enzymes and DNA methylation status in learning and memory paradigms [56].

In terms of epigenetic mechanisms, in addition to histone modifications, we investigated changes in the DNA methylation status of the IEGs *Fos* and *Egr1* in the FS paradigm. Forced swimming resulted in a decrease in DNA methylation at certain 5′-cytosine-phosphate-guanine-3′ (CpGs) within the *c-Fos* and *Egr-1* gene promoters and 5′-untranslated regions specifically in DG neurons; there was no effect in the CA regions of the hippocampus [12]. Furthermore, administration of the endogenous methyl donor *S*-adenosyl methionine (SAM) before the FS challenge reversed the effect of the stressor on the DNA methylation level of the IEGs. Hence, pre-treatment with SAM before forced swimming resulted in an increased DNA methylation of the gene promoters/5′-UTR of *Fos* and *Egr1* and decreased expression of these IEGs specifically within the DG. SAM administration also interfered with the memory consolidation process post-forced swimming, because the rats presented significantly less behavioral immobility than the vehicle-treated animals during the FS re-test 24 h later [12]. Importantly, SAM exerted no effects on the forced swimming-induced formation of H3S10p-K14ac in DG neurons; thus, the methyl donor did not affect the FS-activated signaling pathway required for IEG induction in these neurons [12]. Follow-up experiments revealed that the stressful challenge resulted in increased mRNA expression of *Dnmt3a*, but not *Dnmt3b* or *Tet1* (an enzyme that catalyzes the conversion of 5-mC to 5-hmC, a proposed first step in active DNA demethylation), specifically in the DG. Moreover, our ChIP studies showed an increased binding of DNMT3A to gene promoters of *Fos* and *Egr1* [12]. An increased binding of DNMT3A appears counter-intuitive in the face of decreased DNA methylation after FS. Under conditions of elevated intracellular Ca^2+^ levels, however, it has been shown in vitro that DNMT3A acts as a DNA demethylase [45–47]. As events resulting in IEG induction in the DG neurons are NMDA-R-dependent, elevated Ca^2+^ levels are to be expected in these neurons, potentially favoring DNMT3A to act as a DNA demethylase. Under conditions of elevated SAM levels, despite risen Ca^2+^ levels, the enzymatic activity of DNMT3A may revert to that of a DNA methyltransferase activity [45–47], explaining the enhanced DNA methylation of the *Fos* and *Egr1* gene promoter/5′-UTR region after FS in the presence of elevated SAM [12]. Thus, the DNA methylation status of these IEGs is governed by environmental stimuli (e.g., stress), the availability of the methyl donor SAM, and other physiological factors (e.g., Ca^2+^). Our studies have shown that the forced swimming-induced behavioral immobility response is controlled by GRs, NMDA-Rs, the ERK1/2/MSK1/2-ELK1 signaling pathway, formation of H3K9ac-S10p-K14ac, and DNA methylation status at IEGs in DG neurons, whereby the forced swimming-evoked DNA demethylation plays a critical go-no go role for gene transcription and memory consolidation (Figure 1).

GRs clearly act on neuronal functions via different mechanisms but are themselves subjects of control as well. For instance, and relevant for FS-associated processes, we discovered that GR expression is altered after a FS event as a result of DNA methylation changes and microRNA expression. Within 15 min of this stressful challenge, we found a significant reduction in GR mRNA expression, but not MR mRNA expression, specifically in the DG [59]. We found that forced swimming results in increased DNA methylation of the *Nr3c1* gene associated with an enhanced binding of DNMT3A, which may explain the reduction in gene expression after stress. Furthermore, as it has previously been shown that the microRNA mir-124a can reduce GR mRNA expression in vitro [60], we investigated the expression of this microRNA under baseline conditions and after FS stress in vivo. Forced swimming indeed evoked a significantly increased expression of mir124a in the DG, which was negatively correlated with the expression of GR mRNA expression. Using ChIP we investigated binding of GRs to a putative negative GRE within the *Nr3c1* (GR) gene after FS to determine if GR was capable of suppressing its own expression, but no significant binding in this region was found [59]. These novel observations add to the complexity of regulatory mechanisms controlling GR expression and function in the brain.

## 3 Glucocorticoid Hormone Action at the Genomic Level after Stress

During and after stressful events, GC hormones play an important role in the brain in regulating adaptive physiological and behavioral responses relevant to the stressful challenge [18]. GCs are secreted from the adrenal glands following HPA axis activation. In higher limbic brain structures like the hippocampus and amygdala, and in the prefrontal cortex, GCs play a critical role in the cognitive processing of (psychologically) stressful challenges [3,4,61]. In addition, GCs elicit negative feedback to hypothalamic nuclei (most importantly, the paraventricular nucleus (PVN)) and other parts of the brain to dampen the surge in HPA axis activity after acute stressful events [4,5,61].

The principal brain structure involved in the consolidation of contextual memories associated with such challenges is the hippocampus. It has been known for several decades that, in rodents and humans, GCs are vital for memory consolidation after stressful encounters [4]. It is, however, still unclear exactly how GCs act on the hippocampus to fulfill this function. One notion is becoming increasingly clear though: disruption of GC action is detrimental for brain function as it increases vulnerability for developing mental disorders like major depression, anxiety, and schizophrenia, and possibly neurodegenerative diseases as well.

In view of the scope of this article and the vast GC-action-in-the-brain field, we cannot provide an elaborate account about all aspects here. There have been two significant developments that we wish to address. Our novel finding on the non-genomic interaction of GRs with the NMDA-R/ERK MAPK pathway with epigenetic and gene transcriptional consequences and behavioral implications has been described earlier in this review. The other new data concern the interaction of MRs and GRs with the hippocampal genome after stress in vivo.

As mentioned before, GCs bind to MRs and GRs in the brain, which act as ligand-dependent transcription factors affecting the transcription of GC target genes via interaction with GC response elements (GREs) [62,63]. These corticosteroid receptors can affect transcription in different ways such as directly recruiting chromatin modifying complexes [64]; however, they can also interact with other transcription factors [65] in order to initiate transcription. GRs are ubiquitously localized in the brain, whereas MRs are primarily located in the hippocampus [5,6,66,67]. Hippocampal MRs and GRs are co-localized in pyramidal and granular neurons [7]. GC secretion from the adrenal glands shows two distinct patterns of activity [68]. First, as a result of the pulsatile secretion pattern with varying amplitudes across the day, the baseline levels of GCs follow a circadian rhythm in rodents with low levels in early morning (AM) hours and substantially higher levels in the late afternoon/early evening (PM) at the start of the active phase [69–71]. Second, as a result of exposure to stress, there is a surge in GC secretion, which can be generated at any time of the day, the amplitude of which usually surpasses the circadian-induced rises in the GC secretion [5,66,69].

The occupancy pattern of MRs and GRs under varying circulating GC conditions was published by Reul and de Kloet more than 30 years ago [5]. The occupancy of hippocampal GRs by endogenous GCs (in the rat) strongly depends on the circulating hormone concentration with very low occupancy levels during the early morning and much higher occupancy levels during the evening and after stress [5,66]. In contrast, MRs, due to their very high affinity for binding corticosterone (>10-fold higher binding affinity than that displayed by GRs), were found to be highly occupied by hormones under all physiological (baseline or stress) conditions [5,66]. Based on these early observations, the concept was developed that apparently MRs exert a tonic action of brain function, whereas GRs are involved in neuroendocrine and brain functions associated with elevated GC levels such as negative feedback on HPA axis activity and memory consolidation [3,5,61,72].

Until recently, it was unknown how hippocampal MRs and GRs interact with the genome under baseline and stress conditions in vivo. Previously, pharmacological approaches had been used to study the genomic interaction of these receptors by injecting GCs into adrenalectomized (ADX) rats [73,74]. Using ChIP, we used intact rats to investigate the binding of MRs and GRs to GREs within promoters or intronic regions of the GC target genes *Fkbp5* (FK506-binding protein 5), *Per1* (period 1) and *Sgk1* (serum/glucocorticoid regulated kinase 1) in an extensive time course analysis after FS stress [10]. Binding of both MRs and GRs to these GREs was low in hippocampal chromatin from rats killed under early morning baseline conditions but showed a significant, transient increase after forced swimming with peak levels at 30 min post-stress [10]. Regarding GR, this GRE binding pattern was expected as it dovetailed with the receptor’s occupancy/activation pattern post-stress [66]. In contrast, the observed increase in MR to GRE binding after stress in these target genes was very surprising as we had expected, based on its constant high hormone occupancy levels, that GRE-binding would be high or near-maximal already under early morning baseline conditions; this was clearly not the case [10]. Thus, although MRs are occupied and located in the nucleus [67] under FS conditions, binding of the MR to these GREs was much lower than expected; only after stress a substantial rise in binding was observed. Elevated MR and GR binding levels were also observed under baseline conditions in the evening when GC levels have risen due to the circadian drive [10].

**Figure 3:**
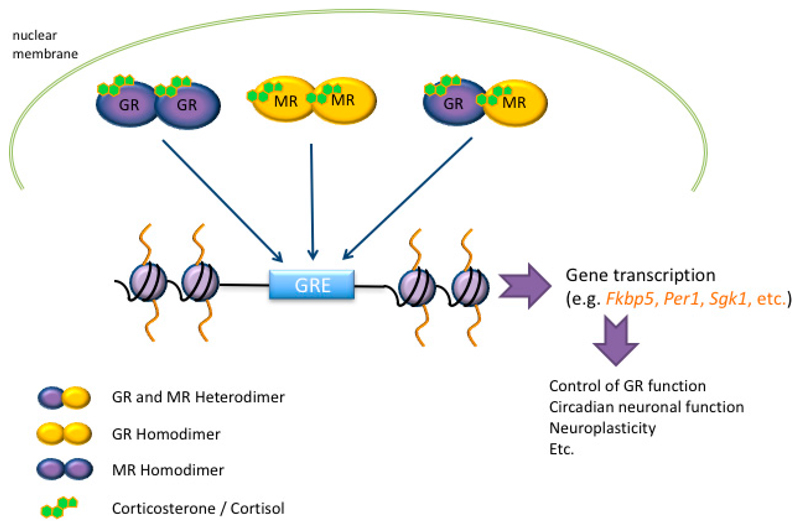
Interaction of mineralocorticoid receptors (MRs) and glucocorticoid receptors (GRs) with glucocorticoid responsive elements (GREs). Natural glucocorticoids (GCs; corticosterone in rodents; cortisol in humans) bind to MRs and GRs, which are ligand-dependent transcription factors that can bind to GREs within GC target genes, like *Fkbp5, Per1* and *Sgk1*, and activate their transcription. MRs and GRs exert these actions through the formation of homo- and/or heterodimers. *Fkbp5:* FK-506 binding protein; *Per1:* Period1; *Sgk1:* Serum/glucocorticoid-regulated kinase 1.

The reason for this remarkable discovery could be that MR binding to GRE is relatively weak, possibly requiring GRs for effective binding to GREs. This idea is supported by transfection studies in vitro [75], which showed that transfection of just MR in monkey kidney COS-1 (CV-1 in Origin with SV40 genes) cells resulted in weak (compared with GR only) DNA-binding and gene transcriptional responses, but if MR and GR were co-transfected then binding and transcription were significantly enhanced beyond levels of the individual receptors. Based on these observations, Trapp et al. [75] proposed the concept that MRs and GRs, in addition to forming homodimers, may also form heterodimers (Figure 3) under conditions of cellular co-localization. Subsequently, heterodimer formation has indeed been determined in cell culture and cell-free systems in vitro [75]; additional references in [10]. Under conditions in vivo, however, heterodimerization of MR and GR had never been shown. We embarked on a series of serial and tandem ChIP studies to investigate MR and GR homo- and heterodimer formation at GREs of GC target genes in the hippocampus under baseline and stress conditions. We discovered that after FS stress, MR and GR form heterodimers (as measured by co-binding) at GREs within the *Fkbp5* and *Per1* genes, but not at the *Sgk1* GRE [10]. In addition, evidence was found for substantial GR homodimer formation after stress, but MR homodimer formation at GREs remained relatively low. These findings support the notion that MR binding per se is rather weak, requiring GR co-binding to strengthen its binding to GREs, but more research is required to strengthen this concept. Alternative mechanisms may be playing a role in restraining access of MRs to GREs under baseline early morning conditions, such as an action of negative steroid receptor co-regulators [10].

These MR and GR ChIP studies using hippocampus tissue have opened a new chapter in the study of the genomic action of GCs in the brain. In addition to the heterodimer discovery, several other key findings were made which have led to adjustments to how we view MR and GR action at the hippocampal genome. We observed that, most clearly after stress, the level of MR and GR binding at the chosen GREs within the classic GC-responsive genes *Fkbp5*, *Per1* and *Sgk1* were markedly different. Overall, levels of binding (i.e., determined as enrichment after ChIP and quantitative polymerase chain reaction (qPCR) analysis) were substantially higher at the *Fkbp5* and *Per1* GREs than at the *Sgk1* GRE [10]. Regarding the *Fkbp5* gene, the GRE referred to here is located within intron 5 of the rat *Fkbp5* gene. There is another GRE within intron 5, upstream of the mentioned GRE, which has been shown in vitro to be inactive and not bind any GRs [76]; indeed, in our study this GRE was relatively inactive in response to FS stress [10]. Thus, it appears that the binding of MRs and GRs to GREs within genes or at gene promoters and enhancer regions is very gene- and GRE-dependent, suggesting that access to GREs is tightly controlled. Further insight into the binding of GREs by MRs and GRs under baseline and stress conditions across the entire rat genome is expected from ChIP studies in combination with next-generation sequencing.

Comparison of different stressors (FS, novelty, and restraint stress), which produce distinct plasma corticosterone responses, revealed that the level of MR and GR binding to GREs within *Fkbp5*, *Per1* and *Sgk1* was not a function of the circulating hormone levels [10]. It appeared that the MR and GR binding levels were similar across stressors and, thus, appeared to require a certain threshold concentration of GCs to produce such receptor GRE-binding levels. An important implication of this observation is that care should be taken when translating circulating GC levels into alleged changes in GC-sensitive functions in the brain.

## 4 Early Life Stress, Epigenetic Dysregulation and Neuropsychiatric Disorders

Psychiatric disorders are heterogeneous and complex, arising from the interaction of many factors such as neurobiology, genetics, cultural background, and life experience. Recent advances within this field have demonstrated that epigenetic mechanisms play an important role in the development and progression of these conditions, especially in early life. It has been well documented that adults who experience childhood stress or trauma have a significantly higher risk of developing a range of mood or other disorders [77,78]. Prenatal adverse environments such as maternal stress can disrupt normal brain development and contribute to neurodevelopmental disorders such as schizophrenia and depression [79,80]. The HPA axis is often found to be dysregulated in psychiatric disorders, particularly in patients suffering from depression, post-traumatic stress disorder (PTSD), and anxiety disorders.

### 4.1 Prenatal Exposure

There is substantial evidence demonstrating that a single acute traumatic experience of a parent can have long-lasting effects on the offspring. This effect has been seen in offspring of mothers who were pregnant and were near to or at the World Trade Centre when it was attacked in 2001 [81]. Previously, mothers and their offspring who both demonstrate reduced levels of cortisol [81] have been linked to an enhanced vulnerability to PTSD [82]. The link, however, between reduced cortisol levels and a predisposition to developing PTSD has been inconsistently reported [83]. The occurrence of PTSD in parents who survived the Holocaust is also associated with lower urinary cortisol excretion in the unexposed offspring (i.e., those conceived after the Holocaust) [82]. This study was later extended by demonstrating that the lower urinary cortisol levels were associated with greater glucocorticoid sensitivity in these offspring [84], which was only evident if the mother had PTSD as a result of living through the Holocaust [85]. To avoid any direct in utero effects, the participants in the study consisted only of offspring who were conceived after the parents had escaped from concentration camps or after liberation. Nevertheless, any (pre-conception) effects on the oocytes of these traumatized women cannot be excluded.

Parents that suffer from PTSD will often pass on an enhanced risk of developing PTSD to their offspring; this is not to deliberately inflict trauma onto the offspring but rather it is thought to be a physiological mechanism to prepare the offspring so that they are able to cope better with the environment they will be subsequently born into. It should be noted that this is still a theoretical concept. There are a number of hypotheses as to how information is passed on through the germ cells; the main theory is the environment provided by the mother in utero. During prenatal development, the epigenome is very susceptible to environmental exposures, and therefore this could be a potential mechanism by which stress-inflicted effects are inherited through the germ cells. This theory has met some scepticism since there is extensive epigenetic reprogramming taking place during embryogenesis to establish cell and tissue-specific gene expression patterns. For instance, normally there is very little variation between tissue-specific methylation patterns [86]; however, there are specific regions within the genome that are more susceptible to variation and these are called metastable epialleles. It is in these regions that establishment of methylation patterns during early development can vary, resulting in variable gene expression and phenotype, but the question is: How do these epigenetic marks escape the reprogramming?

Human studies have shown how early life events impact on the epigenome and persist into adulthood. The Dutch Hunger Winter resulted in individuals being exposed to famine as a consequence of the German occupation towards the end of the Second World War in the winter of 1944–1945. An epigenetic epidemiological study demonstrated that prenatal exposure to famine is associated with lower DNA methylation of the insulin-like growth factor 2 (IGF2) differentially methylated region (DMR) which persisted over 6 decades [87]. Interestingly, this was only observed when exposure occurred during early, but not late, gestation, indicating a critical period for DNA methylation changes to occur. These babies were relatively small when they were born and, later in life, suffered from diseases such as coronary heart disease and presented a two-fold increase in the risk of developing schizophrenia [88].

### 4.2 Post-Natal Exposure

There is a substantial amount of evidence demonstrating that early-life events (post-natal) can potentially have long-term consequences on behavior and stress responsivity that can persist into adulthood. It has been shown that early life stress such as maternal separation in mice can induce histone acetylation that correlates with the activation of synaptic plasticity genes *Arc* and *Egr1* in the hippocampus of the pups [89]. The authors speculated that this adaptation of the hippocampal synaptic circuits occurs in order for the mice to cope with their stressful environment; however, direct evidence is still lacking. The ability to vary phenotype in response to environmental conditions is referred to as phenotypic plasticity. Early postnatal life is a period when the environment can influence emotional and cognitive development. Weaver et al. showed that rat mothers who were more nurturing towards their offspring, as demonstrated by more pup licking (LG) and arched-back nursing (ABN), resulted in a significantly reduced level of DNA methylation along the EGR1 binding site within the hippocampal GR promoter in the pups [90]. The reduction in DNA methylation was associated with an increase in hippocampal GR expression, enhanced glucocorticoid feedback sensitivity, and, as a consequence, resulted in a stronger dampening of the HPA axis response to stress when compared with pups who received low levels of LG and ABN. Accordingly, the offspring which received more maternal care demonstrated decreased hypothalamic corticotropin-releasing factor (CRF) expression and a more modest HPA response to stress. This was also reflected in the pup’s behavioral response to stress, where they displayed less fearful behavior compared with those pups who had received a low level LG and ABN [90]. This study further included a cross fostering experiment, in which they swapped the pups from rat mothers who showed a high level of maternal care with those that displayed a low level of maternal care. This resulted in the pups adopting a similar epigenetic and behavioral profile to the foster mother rather than their biological mother demonstrating how the environment can directly affect the epigenome and phenotype of the offspring. This phenotypic plasticity persisted into adulthood with the foster pups also adopting the high level or low level maternal care of the foster mother. It is interesting to consider that if the conditions experienced by the foetus in utero are resulting in variations in the epigenome, could this be altered by early life postnatal experiences which would persist into adulthood and if so, would they be permanent or can they be reversed?

Current evidence is lacking regarding the mechanism underpinning the persistence of epigenetic marks into adulthood. Possibly, once the epigenetic marks are established, then, most likely, DNA methylation is very stable in adulthood and so persists in mature post-mitotic neurons. There is now, however, ample evidence that the epigenomic marks established early in life through behavioral programming are reversible in the adult brain [91]. Moreover, there is growing evidence demonstrating that epigenetic processes are highly dynamic in the mature post-mitotic neuron and, in fact, are essential for neuronal plasticity [12,18,92].

The evidence that epigenetic mechanisms are involved in cognitive and adaptive responses to stress is continuously growing and presently there are suggestions that the early life epigenetic programming that appears to persist into adulthood may also be able to span generations. Therefore, changes in the environment which can modify the epigenome could potentially be inherited.

### 4.3 Transgenerational Epigenetics

The idea that changes in the ancestral environment can be passed onto descendants is not necessarily new. The developing foetus in utero could be directly affected if the mother were to experience a harsh environment. Another example would be that parental behaviour early in life can govern how the offspring behave as parents later in life. A new theory is now beginning to emerge as to how environmental changes that alter the epigenetic profile of an individual can result in a different phenotype (phenotypic plasticity) that can be inherited across generations. This non-genomic inheritance through the generations is referred to as transgenerational epigenetics, but it has received some scepticism too.

It is important at this stage to define intergenerational and transgenerational transmission. Adult mice that are exposed to an adverse environment will be affected but so will the germ cells; therefore, the subsequent F1 generation would be considered as intergenerational, and only the F2 generation would be transgenerational. In pregnant females (F0 generation), however, intergenerational inheritance will affect the developing fetus’s somatic and germ cells (F1 generation) and the germ cells of the developing fetus (F2 generation); therefore, in order for transgenerational inheritance to be considered, offspring should be studied in the F3 generation. The field of epigenetics remains to be divided over the theory that epigenetics can transgress generations but there is some fascinating research. Studies in mice provided compelling evidence of this phenomenon and, interestingly, the majority of the work has focused on paternal transmission due to the fact that any transgenerational effects can be studied in the F2 generation. Work conducted by Dias et al. using olfactory fear conditioning has suggested that parental traumatic exposure can be inherited in a transgenerational epigenetic manner [93]. The experiment was based on olfactory fear conditioning studies in which male mice (F0) were exposed to acetophenone (an odor that activates the olfactory sensory neuron) and given a foot shock so that in subsequent exposures the mice display fear behavior when presented with this specific odor. This F0 generation was mated with naive female mice and the male F1 offspring was found to display enhanced sensitivity upon presentation of acetophenone. Subsequently, an F2 generation was produced by in vitro fertilization (IVF) with F0 sperm, along with cross fostering studies [93]. The authors suggested that the inheritance of the enhanced sensitivity to acetophenone in subsequent generations was based on the sperm and that this resulted in hypomethylation of the mouse odorant receptor gene *M71* in F0 and F1 generations, which may have led to enhanced gene transcription. The authors concluded the presence of both intergenerational and transgenerational epigenetics. This conclusion raised the question as to how environmental information can get into germ cells. This was explained by the odorants getting into the circulatory system and potentially activating odorant receptors that are expressed in the sperm [93]. Explaining how specific loci can escape the epigenetic reprogramming which occurs after fertilization and then again in the primordial germ cell remains unanswered. It has been shown, however, that some loci associated with metabolic and neurological disorders can be resistant to DNA demethylation [94].

In a further study, chronically social defeated male mice were bred with naive female mice after which their offspring (F1) was assessed for stress-related and anxiety-like behaviors [95]. The offspring from the male mice who experienced chronic social defeat demonstrated an increase in anxiety-like and stress-related behaviors compared with control mice, which was more apparent in the male offspring; an observation that corresponds with those of Dias et al. [93] on olfaction fear conditioning. Interestingly, these observations were not made when offspring was generated using IVF, indicating that the behavioral effects observed were unlikely to be due to inheritance of epigenetic marks [95]. Consequently, there appears to be added complexity in that female mice may be altering their maternal care of the offspring as a result of their exposure to a male mouse with a history of chronic stress [95].

While maternal influences on the stress response have been widely investigated, more studies are now focusing on the potential role of paternal factors. Rodgers et al. [96] showed that chronic paternal stress in both adolescent and adult mice resulted in a significantly reduced HPA axis stress-responsivity [96]. These male mice were exposed to seven different stressors, which were randomized and administered once per day over a 42-day period. The offspring of exposed mice displayed reduced corticosterone responses to acute restraint. Gene set enrichment analyses on the PVN and the bed nucleus of stria terminalis (BNST) demonstrated a global change in transcription patterns which could be due to epigenetic reprogramming, resulting in increased expression of glucocorticoid-responsive genes in the PVN, which is consistent with changes in offspring stress responsivity [96]. The authors also showed that there was an increased expression of nine microRNAs in sperm that were thought to underpin the altered stress responsivity in the offspring. The nine microRNAs were shown to function post-fertilisation and were associated with a reduced HPA axis response as assessed by measuring plasma corticosterone levels after restraint stress. The microRNAs were injected into single cell zygotes, which only resulted in the same stress response phenotype when all nine microRNAs were injected simultaneously. The sperm microRNAs are thought to selectively target maternal mRNA resulting in post-transcriptional silencing of expression. The study did not, however, explore each individual microRNA and their effects on stress responsivity. Nevertheless, the study highlights the putative influence of transgenerational transmission of paternal experiences on the health of the offspring and their resilience [97]. Male mice that had been exposed to unpredictable maternal separation combined with unpredictable maternal stress (MSUS) showed disrupted metabolic and behavioural phenotypes compared with control mice [98]. Deep sequencing analysis of small non-coding RNAs, including microRNAs, present in sperm identified several microRNAs and piRNAs (piwi-interacting RNAs) that were significantly affected by MSUS exposure. Interestingly, these effects (molecular, metabolic and behavioural) could also be induced in male offspring after microinjecting RNAs purified from the sperm of MSUS exposed male mice to wildtype fertilised mouse oocytes. These observations indicate that even in the absence of stress, small, non-coding RNA (sncRNAs) purified from the sperm of stressed mice can transmit the MSUS phenotype to stress-naïve offspring [98].

In a more recent study, further evidence was found for transgenerational epigenetics. F1 male offspring, which had been subjected to chronic and unpredictable maternal separation in early life, were mated with wild-type females that produced an F2 generation. Male mice from the F2 generation were then mated with wild-type females to produce the F3 generation. Increased floating or immobility in the forced swim test and increased immobility in the tail suspension test were seen in the male F1 generation but interestingly not in the females, with the reverse observed in the F2 generation with males not displaying the same behavioral traits as the male F1 generation, while the females did. The F3 generation was assessed and the males once again displayed increased floating in the FS test and increased immobility in the tail suspension test. Furthermore, the unpredictable maternal separation resulted in changes in DNA methylation across a number of candidate genes (e.g., cannabinoid receptor-1 (CB1) and methyl CpG-binding protein 2 (MeCP2)). This research indicates that stress-related behaviors may be transmitted across generations and in sex-specific manner [99].

## 5 Epigenetics as Biomarkers and Therapeutic Treatments

Biomarkers ideally serve to give information about the presence or absence of disease and disease characteristics. The mapping of the complete Human Genome Project (HGP) in April 2003 [100] has assisted in the discovery of novel biomarkers and potential therapeutic targets for treatment of disease. The HGP has also increased our capacity for gene therapy and in identifying single nucleotide polymorphisms (SNPs) where a single nucleotide base in the DNA is mutated and can be associated with a disease. The HGP has assisted with identifying genetic variants that may influence the effectiveness or toxicity of a drug for an individual; this is referred to as pharmacogenomics [101]. Following on from the HGP the Epigenome Project is currently underway, aiming to characterize the epigenome of healthy cell types, tissues, or individuals to obtain “reference” epigenomes. This project is arguably much larger than the HGP due to the different epigenetic modifications and their potential combinations that can dictate gene transcription in a tissue-specific manner. Having a complete epigenome as a reference database, may speed up the production of epigenetic biomarkers and assist in developing new therapeutic treatments. Whilst this is a compelling idea, generating a complete epigenome for any cell, tissue, or animal is a monumental feat that may never be fully completed due to the numerous combinations of epigenetic marks and their continual interaction with the ever-changing environment. There is, consequently, now a rapidly evolving discipline called pharmacoepigenetics that studies the effects of epigenetic factors on the individual variation in responses to drugs [102]. In an ideal world, personalized medicine would encompass different sources of information such as the individual’s genetic and epigenetic make-up, RNA levels, proteins and various other metabolites allowing for a more complete but more complex picture.

The pattern of aberrant DNA methylation changes in cancer is well established, with global hypomethylation accompanied by targeted hypermethylation of some gene promoter CpG islands (CGIs) and, in particular, tumor-suppressor genes [103]. This led to significant interest in finding DNA methylation biomarkers for cancer classification and disease prognosis, with some success [104–106], although it still remains a challenge to separate driver from passenger epigenetic changes.

There have also been advances in identifying aberrant patterns of histone modifications to provide clinical information about cancer [107]. Additionally, studies have identified epigenetic changes associated with other diseases such as lupus [108], diabetes [109], and asthma [110], with potential for therapeutic intervention [111].

Epigenetic treatments are currently being used, with first generation epigenetic pharmaceuticals, such as DNMT and HDAC inhibitors, currently FDA approved for cancer. Despite this, the lack of specificity for individual enzymes and toxicities means more work is needed to refine these shortcomings and improve epigenetic drug discovery. The potential of epigenetic medicine to be combined with conventional medicine to revolutionize the diagnosis and treatment of human diseases is on the horizon.

## 6 Conclusions and Future Perspectives

The stress response, including the consolidation of memories of the stressful event, are essential for an animal’s survival. The process of consolidating stress-related memories involves a number of complex pathways such as the concomitant activation of NMDA-Rs and GRs resulting in the activation of the ERK MAPK pathway, and subsequent epigenetic modifications and gene transcriptional responses. GCs, therefore, play an integral part in producing long-lasting memories through this recently discovered non-genomic mechanism [4,13,27]. Classically, however, GC action through MRs and GRs occurs through genomic mechanisms. Although these mechanisms have been known since the 1980s, it is still unknown which MR- and/or GR-regulated genes are critically involved in stress-related memory formation. Due to advancements in the ChIP technology, the state of knowledge is presently rapidly progressing. Recently, we reported for the first time on the binding of MRs and GRs to GC target genes in the hippocampus after stress in vivo [10] The combination of ChIP with next-generation sequencing will soon lead to the elucidation of the MR- and GR-targeted genes involved in stress-associated learning and memory responses. Furthermore, adding to the complexity of GC action in the brain, our research has shown that the expression of GR is diminished after a stressful challenge, possibly as a result of enhanced DNA methylation and microRNA action.

The long-term impact of epigenetic changes is underscored by the often life-long effects of manipulations inflicted upon the unborn organism in utero or on the newborn during early life. Evidence has been accumulating indicating that epigenetic marks could be transgenerationally transmitted through the germ line. Further research is needed to understand the molecular and epigenetic mechanisms underpinning this process.

The influence of the environment on epigenetic mechanisms is now recognized and is being targeted as a source of potential biomarkers in the diagnosis of various diseases but as a potential target for therapeutic treatment as well. These endeavors are, however, a long way off but may be assisted by advancements in the Human Epigenome Project. This project is of course very ambitious and will be infinitely more complex when compared with the Human Genome Project. With the idea of tailoring medicine for individuals based on their specific epigenetic (and genomic) profile with pharmaco-epigenetics facilitated by advancing technologies, personalized medicine may become a reality.
